# Measurement of Sedentary Behavior—The Outcomes of the Angle for Posture Estimation (APE) Method

**DOI:** 10.3390/s24072241

**Published:** 2024-03-31

**Authors:** Henri Vähä-Ypyä, Pauliina Husu, Harri Sievänen, Tommi Vasankari

**Affiliations:** 1 The UKK Institute for Health Promotion Research, 33500 Tampere, Finland; pauliina.husu@ukkinstituutti.fi (P.H.); harri.sievanen@ukkinstituutti.fi (H.S.); tommi.vasankari@ukkinstituutti.fi (T.V.); 2 Faculty of Medicine and Health Technology, Tampere University, 33014 Tampere, Finland

**Keywords:** accelerometer, measurement, physical activity, sedentary, body posture, cardiorespiratory fitness, body mass index

## Abstract

Hip-worn accelerometers are commonly used to assess habitual physical activity, but their accuracy in precisely measuring sedentary behavior (SB) is generally considered low. The angle for postural estimation (APE) method has shown promising accuracy in SB measurement. This method relies on the constant nature of Earth’s gravity and the assumption that walking posture is typically upright. This study investigated how cardiorespiratory fitness (CRF) and body mass index (BMI) are related to APE output. A total of 3475 participants with adequate accelerometer wear time were categorized into three groups according to CRF or BMI. Participants in low CRF and high BMI groups spent more time in reclining and lying postures (APE ≥ 30°) and less time in sitting and standing postures (APE < 30°) than the other groups. Furthermore, the strongest partial Spearman correlation with CRF (r = 0.284) and BMI (r = −0.320) was observed for APE values typical for standing. The findings underscore the utility of the APE method in studying associations between SB and health outcomes. Importantly, this study emphasizes the necessity of reserving the term “sedentary behavior” for studies wherein the classification of SB is based on both intensity and posture.

## 1. Introduction

Sedentary behavior (SB) is defined as any waking behavior characterized by a low energy expenditure, ≤1.5 metabolic equivalents (METs), while in a sitting, reclining, or lying posture [1]. Stationary behavior refers to any waking behavior while lying, reclining, sitting, or standing, with no ambulation, irrespective of energy expenditure [1]. Sitting is a position in which one’s weight is supported by one’s buttocks rather than one’s feet and one’s back is upright [1]. Reclining is a body position between sitting and lying, and lying refers to being in a horizontal position on a supporting surface [1]. Standing is a position in which one is maintaining an upright position while supported by one’s feet [1]. Energy expenditure, heart rate, skeletal muscle blood flow, and contractile activity are higher during sitting compared to reclining but lower compared to standing and physical activity (PA) of any intensity [2].

Excessive and uninterrupted SB may lead to insulin resistance, vascular dysfunction, shift in substrate use towards carbohydrate oxidation, shift in muscle fiber from oxidative to glycolytic type, reduced cardiorespiratory fitness, loss of muscle mass and strength, bone loss, increased total body fat mass and visceral fat depot, higher blood lipid concentrations, and increased inflammation [2,3]. Additionally, total SB time is negatively associated with cardiorespiratory fitness (CRF) [4,5], and the association between SB and all-cause mortality is more pronounced among physically inactive people [6,7]. From a physiological perspective, there are impacts of SB on physiological responses that resemble those of physical inactivity, i.e., too little exercise. Even though such effects are similar, high volumes of SB can confer adverse physiological effects even in the presence of large volumes of aerobic and/or resistance exercise [2].

SB can be measured with self-reports or device-based methods [8]. Self-reported measures can provide more contextual information than device-based methods, but they generally underestimate sedentary time when compared to device-based measures [9,10]. The discrepancy between the self-reported and device-measured sitting time is differently biased by several variables, most noticeably by age, body mass index (BMI), and physical work demands [11]. Device-based methods can be typically classified as accelerometer-based or inclinometer-based methods. Accelerometer-based sensors use the intensity of movement-generated accelerations to estimate energy expenditure during daily life. whereas low-intensity activities, below a specified acceleration threshold, are classified as SB [12]. Inclinometer-based sensors measure the inclination of body part(s) to estimate postural information such as standing, sitting, and lying [12]. Typically, the inclinometer-based sensors are thigh-worn, where vertical thigh orientation typically indicates a standing posture and horizontal thigh orientation indicates a sitting posture [13]. However, this approach has some challenges to distinguish lying from sitting [14]. Thus, with device-based methods, the SB measurement can be based solely on intensity (≤1.5 METs) or a combination of low intensity (≤1.5 METs) with a seated, reclining, or lying posture [15].

Hip-worn accelerometers have been known for their ability to provide useful information about the association between PA and health outcomes [16]. However, limitations arise from the inability of inclinometer algorithms to accurately identify lying, sitting, and upright activities [17]. Consequently, researchers should exercise caution when considering the utilization of the inclinometer function of hip-worn accelerometers, particularly in studies emphasizing the duration of SB [17]. The inclinometer function is sensitive to factors such as body shape, because the amount and distribution of body fat can alter the tilting angles of the inclinometer [18,19]. Thus, BMI has an additional impact on the measurement of sitting time using the inclinometer function of accelerometers. Individuals with higher BMI tend to sit more, but BMI is associated with body shape and composition, which may compromise the ability of hip-worn accelerometers to accurately detect body postures [18]. The recent developments in the field of hip-worn accelerometer algorithms have made it possible to quantify SB as per the proper definition. Both the angle for postural estimation (APE) method [20] and convolutional neural network hip accelerometer posture (CHAP) method have shown over 90% accuracy in classifying body postures [21,22]. The APE method is based on two concepts: the Earth’s gravity vector is constant, and the body posture during walking is upright. The accelerometer orientation, in terms of the gravity vector during walking, is taken as the reference, and the APE is determined from the concurrent accelerometer orientation in relation to the reference vector [20]. The APE method has also been evaluated and validated by others [23]. The CHAP is a machine learning model, which combines a convolutional neural network with a bi-directional short-term memory network and a SoftMax output layer to predict sitting or non-sitting posture from raw hip-worn acceleration data [21]. The developers of the CHAP method have found that the measured postural component of SB is more strongly associated with obesity variables than the measured stationary behavior [24].

We hypothesized that individuals with lower CRF and higher BMI values tend to engage in more SB and spend less time in standing and PA compared to those with higher CRF and lower BMI values. For the APE method to be considered valid for population measurements, it should demonstrate the ability to discriminate between sedentary and standing times in groups with different background variables. Additionally, this study aimed to define the recommendations for the use of APE method in SB measurements.

## 2. Materials and Methods

This study is based on a subsample of the population-based FINFIT 2017 and 2021 studies [25,26]. These are cross-sectional population studies based on a stratified random sample of 20–69-year-old Finnish men and women from seven city-centered regions. The data collection of the FINFIT 2017 study was conducted between September 2017 and March 2019 and the FINFIT 2021 study between September 2021 and June 2022. The coordinating ethics committee of The Regional Ethics Committee of the Expert Responsibility Area of Tampere University Hospital gave ethical approval for the studies (R17030 and R21050). 

At the health examination of the study, participants’ height and weight were measured, and they performed a 6 min walk test (6MWT) [25,26]. In short, the participants were asked to walk back and forth along a 15 m walking track as fast as possible for six minutes. Their heart rate was recorded with a heart rate monitor (Polar M61, Polar Electro, Kempele, Finland). For men, the maximum oxygen uptake (VO_2_max) was predicted from the walking distance in six minutes, age, body mass index (BMI), heart rate at the end of the test, and height [27]. For women, the heart rate was not a statistically significant predictor, and the prediction was based on the walking distance in six minutes, body mass, and age [27]. These predictors explained 82% (standard error of estimate (SEE) = 3.6 mL/kg/min) of the variation in the measured VO_2_max in men and 79% (SEE = 3.5 mL kg/min) in women [27].

**Figure 1:**
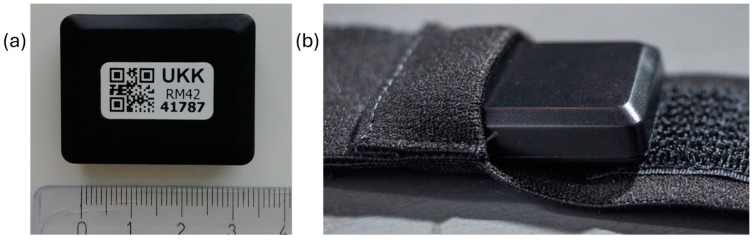
(**a**) The dimensions of the UKK RM42 accelerometer are 35 × 27 × 9 mm, with a weight of 9.3 g. (**b**) For waking time use, the UKK RM42 is pushed into the bottom of a tight, elastic belt-pocket.

At the health examination, the participants also received a triaxial accelerometer (UKK RM42, UKK Terveyspalvelut Oy, Tampere, Finland) to be used for seven consecutive days all the time (24/7) except during showers and other water activities. The accelerometer was attached to a flexible belt with an instruction to wear the belt so that the accelerometer was on the right hip during waking hours and on the nondominant wrist during the time in bed (Figure 1). The participants also received a sleep diary, where they marked the times when they went to bed for sleeping and woke up.

The acceleration signal was collected at a 100 Hz sampling frequency, ±16 g acceleration range (g is Earth’s gravitational constant), and 0.004 g resolution. The accelerometer was initialized so that it started collecting data if the absolute difference between a reference value and the incident acceleration exceeded 187.5 mg (mg denotes milligravity) in any axis and, within the next 5 s, the difference exceeded 500 mg in any axis; if not, the accelerometer returned to quiescent state [28]. Whenever the raw acceleration in any axis exceeded the above-mentioned limits, the reference values were updated with the coincident acceleration values. The period was considered as non-wear time if continuous quiescent time was longer than 120 min [29]. After the one-week measurement, the accelerometers and sleep diaries were returned, and the raw data were stored on a hard disk for further processing and analysis. The analysis was performed with the Microsoft Excel Visual Basic for Applications program (2016; Microsoft Corporation, Santa Rosa, CA, USA).

The raw accelerometer data were analyzed in 6 s epochs [30]. For each epoch, the mean amplitude deviation values of the resultant acceleration signal (MAD) and of the acceleration signal in each orthogonal direction (MADxyz) were calculated using Equations (1)–(3) [30]. In the equations, r_i_ represents the magnitude of the acceleration components x_i_, y_i_, and z_i_; N is the number of samples in the epoch; and R_ave_, X_ave_, Y_ave_, and Z_ave_ are the mean acceleration values of the epoch.

(1) $$r_{i}=\sqrt{x_{i}^{2}+y_{i}^{2}+z_{i}^{2}}$$

(2) $$MAD=\frac{1}{N}\sum_{i=1}^{N}\left|r_{i}-R_{ave}\right|$$

(3) $${MAD}_{xyz}=\frac{1}{N}\sqrt{{\left(\sum_{i=1}^{N}\left|x_{i}-X_{ave}\right|\right)}^{2}+{\left(\sum_{i=1}^{N}\left|y_{i}-Y_{ave}\right|\right)}^{2}+{\left(\sum_{i=1}^{N}\left|z_{i}-Z_{ave}\right|\right)}^{2}}$$

The epoch-wise acceleration values were converted to METs using Equations (4)–(6) [30].

(4) MET=1.0000+0.0223·MAD  (MAD<91.5 mg)

(5) MET=2.1488+0.0093·MAD  (91.5≤MAD<500 mg)

(6) $$MET=0.4027+0.0107\cdot MADxyz\;\left(MAD\geq 500\;mg\right)$$

If the MAD value was below 91.5 mg, Equation (4) was used; for MAD values between 91.5 mg and just under 500 mg, Equation (5) was used; otherwise, Equation (6) was used. The accuracy of estimation for intensities typical for walking is approximately 0.45 MET and for running it is 1.58 METs [31].

The distribution of the estimated MET values is presented in a histogram by dividing the data into nine equally wide bins. The bin width is 1.5 METs, with the bins covering the range from less than 1.5 METs to over 12 METs. Subsequently, the mean daily time spent within each MET bin was calculated for each participant. The lowest MET bin, from l to <1.5 METs, corresponds to the stationary behavior; the second lowest bin, from 1.5 to <3 METs, light PA; bins from 3 to <4.5 METs and from 4.5 to <6 METs, moderate PA; bins from 6 to 7.5 METs and from 7.5 to <9 METs, vigorous PA; and bins from 9 to <10.5 METs, from 10.5 to <12 METs, and >12 MET, very vigorous PA [32]. 

The epochs with a MET value less than 1.5 (i.e., MAD value less than 22.5 mg) were further analyzed with the APE method [20]. The APE denotes the angle between the reference vector and the epoch-wise device orientation vector (ê), which contains the measured axis-specific mean values for the given 6 s epoch and is set by Equation (7).

(7) $$\hat{e}=\frac{e}{\left|e\right|}=\frac{x_{ave}}{\sqrt{x_{ave}^{2}+y_{ave}^{2}+z_{ave}^{2}}},\frac{y_{ave}}{\sqrt{x_{ave}^{2}+y_{ave}^{2}+z_{ave}^{2}}},\frac{z_{ave}}{\sqrt{x_{ave}^{2}+y_{ave}^{2}+z_{ave}^{2}}}$$

The accelerometer orientation during walking was used as the reference vector for the APE method and it had to be set before the calculation of postural orientation was possible. The recognition of walking was based on the intensity of activity step rate and movement steadiness. The MAD range for normal walking is 150–350 mg, the number of steps during a 6 s epoch is 8–13, and the ratio of MADxyz to MAD is less than 1.6. Each time, when the previous parameters of the examined epoch were within the normal walking limits, vector ê was set as a new reference vector for the upright posture (û). 

The APE is the angle between the vectors ê and û and is calculated using Equation (8).

(8) $$APE={cos}^{-1}\hat{e}\cdot \hat{u}$$

The optimal cut-point of APE to separate sitting from standing was previously found to be 11.6°, with individual values ranging from 11.4° to 14.2° [20]. In the validation study [20], lying was separated from sitting with absolute accuracy, the highest APE value for sitting activities was 55.9°, and the lowest APE value for lying was 73.9°. APE values are typically higher than 30° for sitting on a sofa and less than 30° for sitting at a desk. The APE method relies on the detection of walking and there is no need to control for the exact orientation of the accelerometer or to know it a priori. The detection of walking provides information about how the accelerometer was attached to soft and compliant tissue. The primary concern lies in proper accelerometer usage, adhering to its intended function, and ensuring a sufficiently firm fixation [20]. 

**Figure 2:**
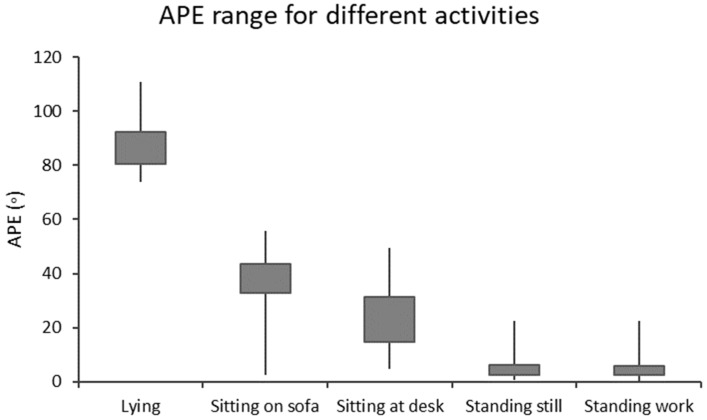
The angle for postural estimation (APE) boxplot for different types of stationary behaviors [20]. The boxes contain 25% and 75% percentiles, and the whiskers are the minimum and maximum values.

In the present study, the stationary time is presented as a histogram by dividing the data accordingly with the APE values into 18 bins with 5° intervals, which covered the APE values from less than 5° to at least 85°. The bins of APE below 5° and APE from 5° to <10° correspond to standing; the bins from APE 10° to <15° can be considered as a transition between standing and sitting; the bins from 15° to <30° correspond to sitting at a desk; the bins from 30° to <55° correspond to sitting on sofa; the bins from 55° to <75° can be considered as a transition between sitting and lying; and the bins upwards from 75° can be considered as lying [20]. Figure 2 shows the ranges of APE values corresponding to various stationary behaviors. 

The criteria for adequate accelerometer data collection were based on the following principles. Days with a complete 24 h wear time and a minimum of 10 settings of the reference vector indicating an upright posture were selected for analysis. Furthermore, to minimize intra-individual variation, a monitoring period of at least four days was required for inclusion in the analyses [33]. A total of 3826 participants completed the 6MWT, with 3587 of them wearing an accelerometer continuously for at least four days over a one-week data collection period. Among these participants, 3475 individuals (1397 men and 2078 women) met the above requirement of daily reference vector settings.

In addition, the influence of CRF or BMI on the times spent in different APE bins was evaluated by grouping the participants into thirds according to their VO_2_max (low, mid, and high) using respective tertiles, and into three categories according to their BMI (low, mid, and high). The low BMI category had BMI values less than 25 kg/m^2^, the mid BMI category had BMI values from 25 kg/m^2^ to 30 kg/m^2^, and the high BMI category had BMI values higher than 30 kg/m^2^. Participants were weighted by the sample size in the five age groups of men and women: 20–29 years, 30–39 years, 40–49 years, 50–59 years, and 60–70 years (nine participants had already reached the age of 70 years by the time they took part in the tests). It was assumed that individuals in the age groups are equally distributed according to sex at the population level. The present analyses were conducted for the weighted data. The differences between the time in the MET and APE bins in the CRF thirds and BMI categories were tested separately for men and women using the independent-samples Kruskal–Wallis test with a post-hoc Dunn’s test adjusted by the Bonferroni correction for multiple tests. Partial Spearman correlations controlled for age, age^2^, and sex were calculated to quantify the strength of association between the times spent in the bins and CRF and BMI. The significance level was set at *p* < 0.05. Additionally, sex-specific hour-by-hour accumulated times of stationary behaviors with APE less than 30° and at least 30° were analyzed for weekdays and weekend days. All statistical analyses were conducted using IBM SPSS version 29.0 (IBM Corp. Armonk, NY, USA). 

## 3. Results

Table 1 shows the number of participants in the CRF thirds and BMI categories broken down by sex and age groups. Most participants in the youngest age group belonged to the high CRF third, and most in the oldest age group to the low CRF third. However, within each age group there were participants from every CRF third. The low BMI category (BMI < 25 kg/m^2^) was the largest for each female age group and for the two youngest male age groups.

Table 2 shows the mean, SD, and ranges of VO_2_max, age, and BMI in the CRF thirds and BMI categories broken down by sex. The participants’ mean age and BMI increased with decreasing CRF. Similarly, the participants’ mean age increased and VO_2_max decreased with increasing BMI. However, high age or BMI did not prevent some individuals belonging to the high CRF third.

**Figure 3:**
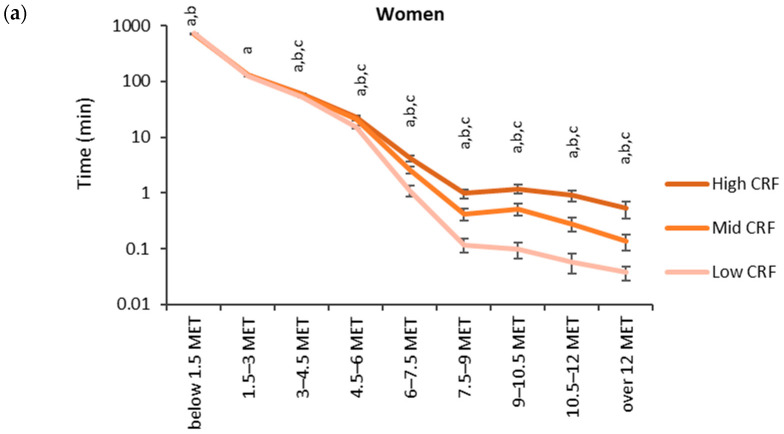

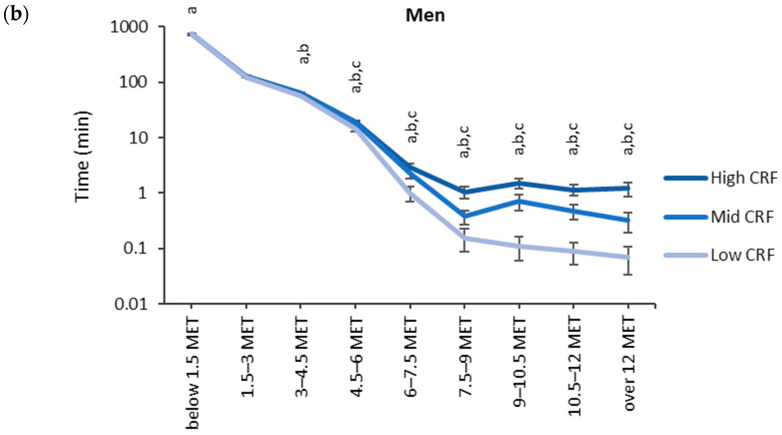
Accumulated times in different MET bins for the CRF thirds in (**a**) women and (**b**) men. The time on the y-axis is presented in the logarithmic scale, and the x-axis shows the MET bins. The letter “a” denotes a statistically significant difference (*p* < 0.05) between the high and low CRF thirds, “b” between the mid and low CRF thirds, and “c” between the high and mid CRF thirds.

The accumulated time in the MET bins broken down by the CRF thirds is shown in Figure 3. Each participant had at least one measured epoch up to the 4.5–6 MET bin and 62% of participants had at least one epoch in the bin corresponding to over 12 METs. The highest accumulated time was observed in the lowest MET bin. In the low CRF third of women, the accumulated time (730 min) in the lowest MET bin was significantly higher than that for the mid (720 min) and high (720 min) CRF thirds. For men, the accumulated time in the lowest MET bin was significantly higher for the low CRF third (742 min) than that for the high CRF third (728 min). From 1.5 METs upward, the mean accumulated time in the MET bins was the highest in the high CRF third and the lowest in the low CRF third. There were statistically significant differences between each CRF third from 3.0 METs upwards for women and from 4.5 METs upwards for men. 

**Figure 4:**
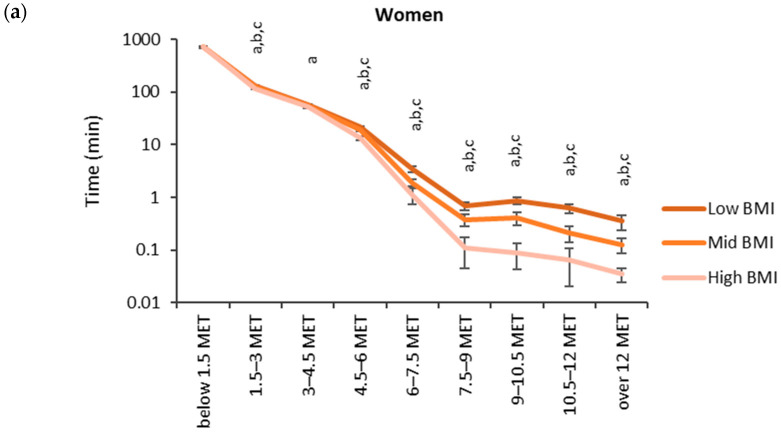

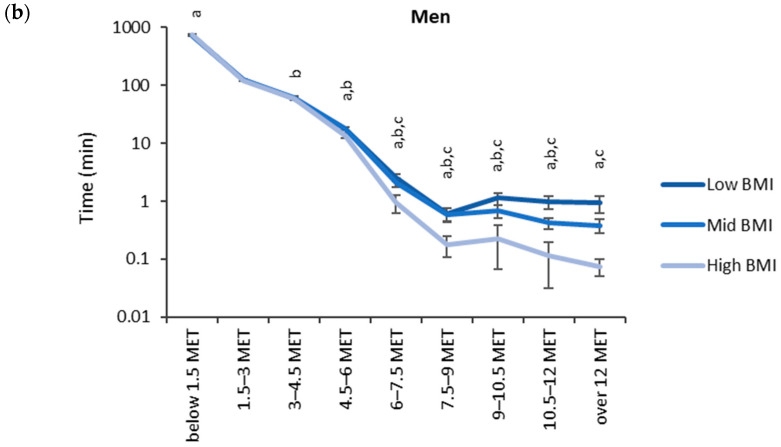
Accumulated times in different MET bins for the BMI categories in (**a**) women and (**b**) men. The time on the y-axis is presented in the logarithmic scale, and the x-axis shows the MET bins. The letter “a” denotes a statistically significant difference (*p* < 0.05) between the high and low BMI category, “b” between the mid and low BMI category, and “c” between the high and mid BMI category.

The accumulated time in the MET bins for the BMI categories (low: <25 kg/m^2^, medium: 25–30 kg/m^2^, high: >30 kg/m^2^) is shown in Figure 4. The highest accumulated times were observed in the lowest MET bin (<1.5 MET). For men, the accumulated time in the lowest MET bin was significantly higher for the high BMI category (749 min) than for the low BMI category (737 min). From 4.5 METs upward, the mean accumulated time in the MET bins was the highest for the low BMI category and the lowest for the high BMI category. There were statistically significant differences between each BMI category from 4.5 METs upwards for women and 6.0 METs upwards for men. 

**Figure 5:**
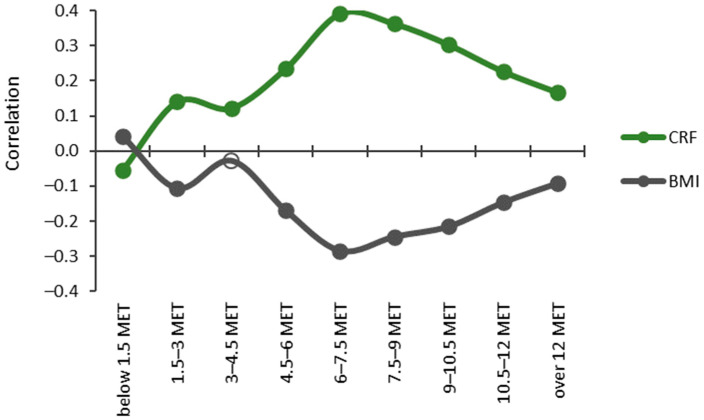
Partial Spearman correlations controlled for age, age^2^, and sex between cardiorespiratory fitness (CRF) and body mass index (BMI) with MET bins. The y-axis shows the correlation coefficient, and the x-axis shows the MET bins. Significant correlations (*p* < 0.05) are indicated with a closed circle, and non-significant with a hollow circle.

The relationship between the CRF thirds and BMI categories and accumulated times in the MET bins was inverted when the MET values rose over the 1.5 MET limit (Figure 5). The accumulated time in the lowest MET bin (<1.5 MET) had a significant (*p* < 0.05) positive correlation with BMI and a negative correlation with CRF. The bins from 3 METs upwards showed a significant positive correlation with CRF and the bin 1.5–3 MET, and the bins from 4.5 MET had a significant negative correlation with BMI. For CRF, the strongest correlation (r = 0.391) was found for the 6–7.5 MET bin and the only negative correlation (r = −0.054) was found for the bin below 1.5 METs. For BMI, the strongest correlation (r = −0.284) was found for the bin of 6–7.5 MET, and the only positive correlation (r = 0.043) was found for the bin below 1.5 METs. 

**Figure 6:**
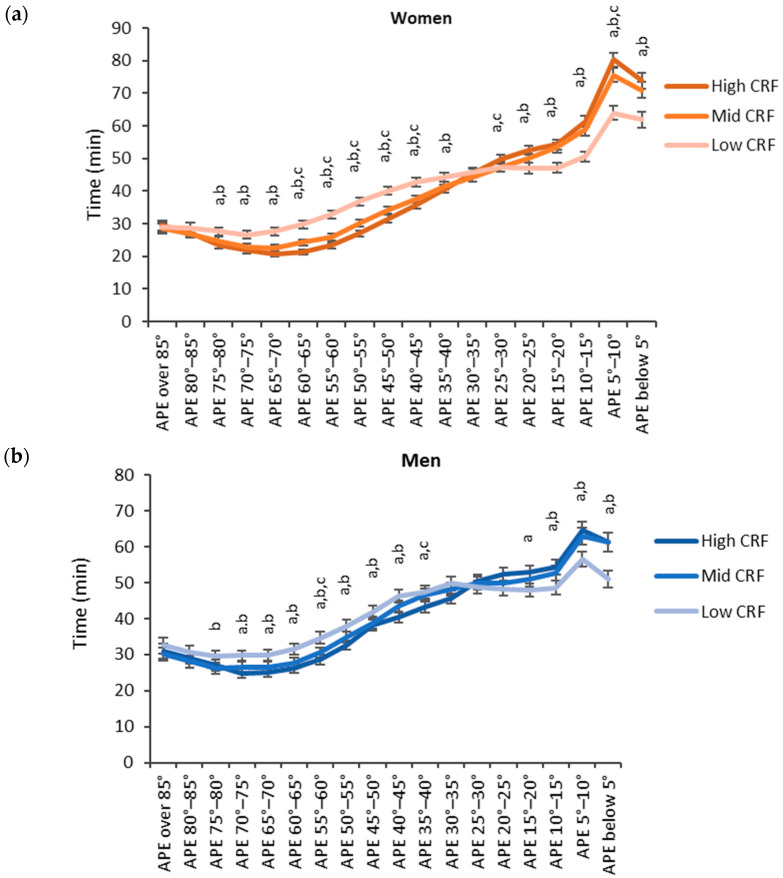
Accumulated times in different APE bins for the CRF thirds in (**a**) women and (**b**) men. The y-axis shows the accumulated time in the bin and the x-axis shows the APE bins. The APE bins values from left to right correspond to lying, reclining, sitting, and standing. The letter “a” denotes a statistically significant difference (*p* < 0.05) between the high and low CRF thirds, “b” between the mid and low CRF thirds, and “c” between the high and mid CRF thirds.

The accumulated time in the APE bins was analyzed for epochs with the MET value less than 1.5 METs Every participant had at least one measured epoch for APE bins between 0° and 85°. For the APE bin over 85°, two participants did not have any measured activity. The highest accumulated times were measured for the two bins with APE values less than 10° (Figure 6). For both sexes, the low CRF third accumulated a significantly lower time for the APE bins less than 15° than the mid or high CRF thirds. Correspondingly, the low CRF third accumulated a significantly higher time for the APE bins between 40° and 75° than the mid and high CRF thirds. 

**Figure 7:**
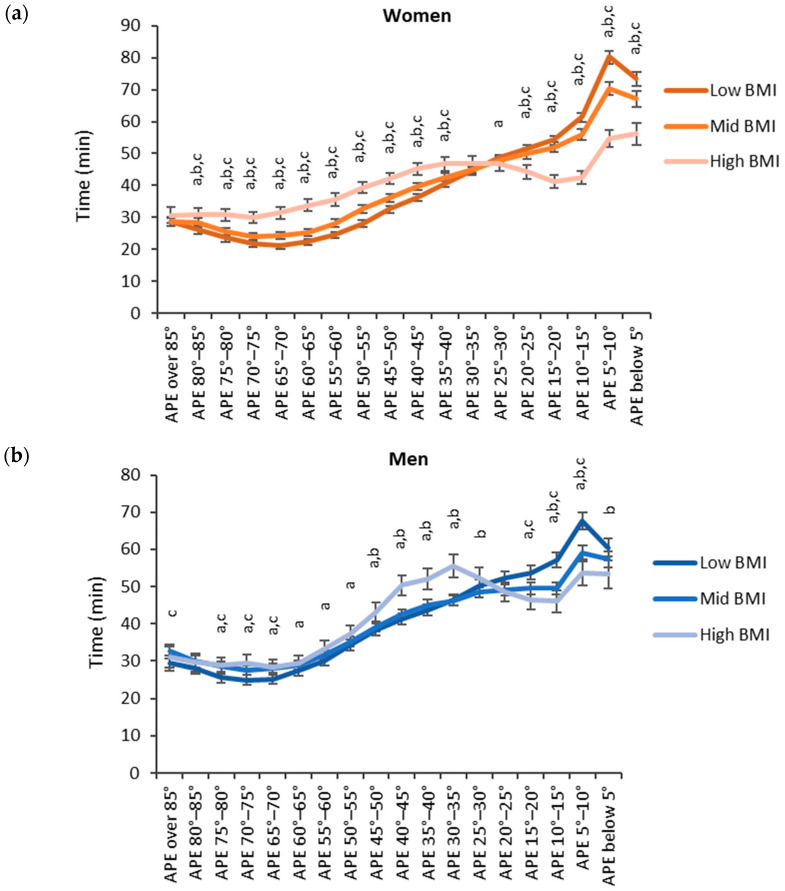
Accumulated times in different APE bins for the BMI categories in (**a**) women and (**b**) men. The y-axis shows the accumulated time in the bin and the x-axis shows the APE bins. The APE bins values from left to right correspond to lying, reclining, sitting, and standing. The letter “a” denotes a statistically significant difference (*p* < 0.05) between the high and low BMI category, “b” between the mid and low BMI category and “c” between the high and mid BMI category.

Regarding BMI, participants with low BMI accumulated more time in the lower APE bins and participants with high BMI accumulated more time in the higher APE bins (Figure 7). For women, the high BMI category (BMI over 30 kg/m^2^) accumulated the significantly highest time and the low BMI category (BMI below 25 kg/m^2^) accumulated the significantly lowest time with the APE bins between 35° and 85°. For the APE bins between 0° and 25°, the results were reversed, and the high BMI category accumulated the significantly highest time and the low BMI category accumulated the significantly lowest time. For men, the results were similar but a significant difference between each category was observed only for the APE bins between 5° and 15°.

**Figure 8:**
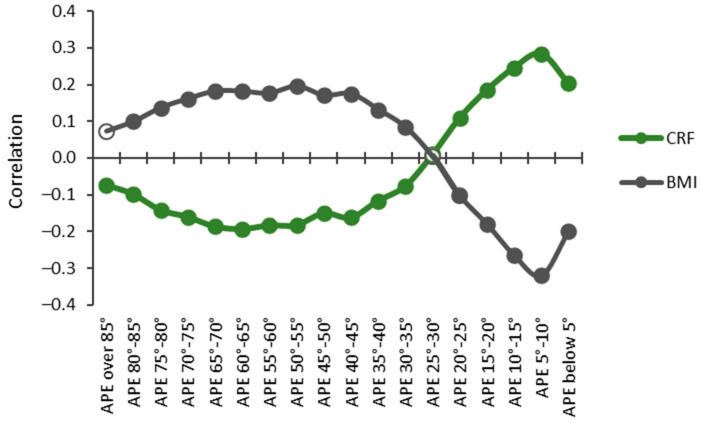
Partial Spearman correlations controlled for age, age^2^, and sex between cardiorespiratory fitness (CRF) and body mass index (BMI) with APE bins. The y-axis shows the correlation coefficient, and the x-axis shows the APE bins. Statistically significant (*p* < 0.05) correlations are indicated with a closed circle, and non-significant with a hollow circle.

The relationships between CRF and BMI and accumulated times in APE bins were opposite for high and low APE values (Figure 8). The accumulated time in the APE values less than 25° had a significant (*p* < 0.05) positive correlation, whereas the time in APE values over 30° had a significant negative correlation with CRF. The strongest positive correlation (r = 0.284) with CRF was found for the APE values between 5° and 10°, and the strongest negative correlation (r = −0.193) was found for the APE values between 60° and 65°. The results were reversed with BMI, and significant negative correlations were found for the APE values less than 25° and positive correlations for the APE values between 30° and 85°. The strongest positive correlation (r = 0.196) was found for the APE values between 60° and 65° and the strongest negative correlation (r = −0.320) for the APE values between 5° and 10°. 

**Figure 9:**
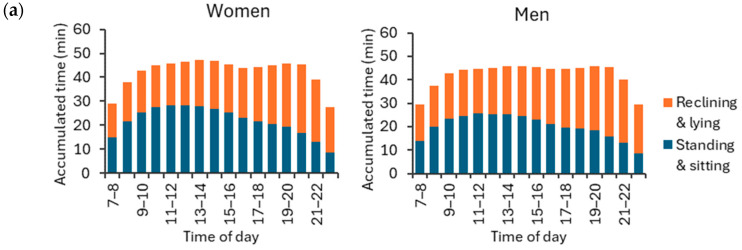

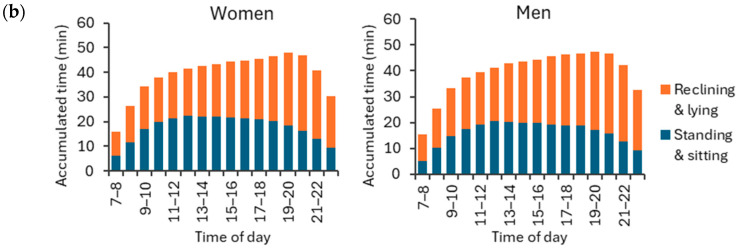
Accumulated hour-by-hour stationary behavior times for APE values at least 30° (reclining and lying) and APE values less than 30° (standing and sitting) during (**a**) weekdays and (**b**) weekend days for women and men. The y-axis shows the accumulated time hour-by-hour, and the x-axis shows the time of the day. The rest of the hourly activities were either physical activity or sleep.

Both women and men accumulated the highest hourly amount of standing and sitting (APE < 30°) between 11 a.m. and 12 p.m. on weekdays, and between 12 p.m. and 1 p.m. on weekend days (Figure 9). As to reclining and lying (APE ≥ 30°), both men and women exhibited the highest hourly amount between 8 p.m. and 9 p.m. on both weekdays and weekends. The peak hourly mean standing and sitting time was 28.5 min for women and 25.0 min for men during weekdays, and 20.6 min for women and 22.2 min for men during weekend days. Correspondingly, the peak mean reclining and lying time was 28.6 min for women and 29.4 min for men during weekdays, and 30.5 min for women and 31.0 min for men during weekend days. 

## 4. Discussion

In this study, PA (>1.5 METs) time was positively associated with CRF and inversely associated with BMI. Conversely, the time spent in stationary behaviors (≤1.5 METs) was associated with a lower CRF and a higher BMI. However, diverse behaviors within stationary behaviors yielded contrasting outcomes. Activities associated with the APE values categorized as lying and reclining showed adverse outcomes, while those categorized as standing indicated positive outcomes. The APE values corresponding to a sitting posture behaved as a transitional zone, where a significant positive outcome transitioned to a non–significant outcome. This study highlights the importance of accurately classifying body postures during stationary activities in understanding their distinct implications on health outcomes.

Energy expenditure is known to be elevated during sitting compared to reclining, yet it remains lower than that observed in a standing position or during PA of any intensity [2]. In this study, the time spent standing (i.e., the APE values between 5° to 10°) had more beneficial association (r = −0.320) with BMI than any other activity. Additionally, surprisingly, the beneficial association (r = 0.284) between the time spent in the APE values between 5° and 10° and CRF was higher than that of the time spent in moderate PA intensities. Only the time spent in vigorous PA intensities had a more positive association with CRF than the time spent in the APE values between 5° and 10°. In general, participants with a higher CRF or a lower BMI spent more time in APE values typical for standing and sitting than those with a lower CRF or a higher BMI. Correspondingly, participants with a higher BMI or a lower CRF spent more time in APE values typical for reclining and lying positions than those with a lower BMI or a higher CRF. 

The findings regarding accumulated SB and PA time in this study are in accordance with respective global guidelines, which emphasize the importance of increasing PA levels and reducing sedentary time [34]. In addition to lower SB time and higher PA time, increased standing time was associated with positive outcomes. It is likely that standing alone does not directly lead to higher CRF or lower BMI values. Rather, individuals who are more physically fit with lower BMIs tend to adopt more upright postures in their daily activities. 

The findings of the present study suggest that SB should be analyzed in more detail. Specifically, the APE values exceeding 30°, indicative of reclining and lying postures, exhibited the most adverse associations with CRF and BMI. Conversely, the APE values less than 15°, indicating standing and the transitional zone between sitting and standing, had the most beneficial association with CRF and BMI. The association with CRF shifts from positive to negative, and that with BMI shifts from negative to positive, for the APE values within a 15° to 30° range, which is characteristic of sitting postures. It is possible that these APE values occur during breaks or interruptions in exercise activities, such as gym workouts or ballgame sessions. 

Additionally, standing and sitting (APE < 30°) were more prevalent during the midday hours, while reclining and lying (APE ≥ 30°) were more common during the evening hours. Mentally active and passive forms of SB might have opposing relationships with health outcomes [8], which has been observed in studies comparing work and leisure time. Reduced SB during leisure time and increased SB during working time have positive associations with health outcomes [35,36]. Therefore, the difference between the reclining and sitting observed in the present study may be attributed to the distinct contexts of SB. It is likely that the sitting time may have accumulated, besides breaks or interruptions in exercise activities, more from office-related activities, while the reclining time may be more representative of leisure-time activities such as watching TV. 

The contextual variation in SB during different activities highlights the importance of considering not only the total sedentary time, but also the specific behavioral context, as it may influence the impact of SB on health outcomes. The present study is limited to a device–based measurement of physical behavior. While these methods afford greater accuracy in capturing sedentary time, supplementing them with self-report data could have provided valuable insights into typical sitting and reclining scenarios. [37]. Additionally, device-based measurements may struggle to accurately categorize active non-ambulatory activities, such as squatting, kneeling, and ground sitting, which all involve muscle activity to maintain body balance [38].

The relationship between replacing SB with standing or PA and its impact on CRF is complex and influenced by various factors. The mechanisms through which SB may affect CRF are not totally understood [4]. For instance, during a 6–month intervention using the MAD and APE methods, reduced SB without adding moderate to vigorous PA did not lead to improvements in CRF [39]. Therefore, the variations in times spent within different PA intensity bins, as observed in the present study, may play a more significant role in influencing CRF and could potentially counterbalance the negative effects of SB [40].

Replacing SB with standing involves greater postural control and muscle activity, which can contribute to positive health outcomes [41]. During a 6-month intervention aiming to replace SB with standing or PA measured with the MAD and APE methods, the insulin sensitivity increased among participants who decreased the SB time [42]. Additionally, during a 3-month intervention, a reduced total SB time conferred beneficial effects on several cardiometabolic risk markers in adults [43]. Despite the lower energy expenditure associated with SB compared to standing, reducing SB without concurrently increasing moderate to vigorous PA may not be an adequately effective strategy for reducing adiposity, such as BMI, waist circumference, or body fat content [2]. This suggests that the positive effects of reduced SB on metabolic health may be more pronounced when accompanied by an increased engagement in high-intensity PA.

Prolonged and uninterrupted SB leads to adverse health effects, and evidence suggests that breaking up the SB time every 20–30 min can be beneficial for health [2,44]. Cross-sectional studies utilizing the MAD and APE methods have shown that individuals with cardiovascular disease or at high risk of those had the highest mean daily number of SB bouts, particularly those lasting for more than 10 min [45]. Moreover, these individuals exhibited a larger waist circumference [46]. Conversely, a higher number of daily sit-to-stand transitions and standing bouts lasting from 30 s to 5 min were both associated with a smaller waist circumference [46]. In previous studies, SB was defined as MAD values less than 22.5 mg and APE values higher than 11.6°. These results underscore the importance of considering not only the total duration of SB, but also the pattern of interruptions and transitions, as they may have distinct implications for health outcomes.

Based on the present findings, there appears to be a rationale for refining the categorization of stationary behaviors in population studies utilizing the MAD and APE algorithms. Specifically, it is proposed to further differentiate sitting into two subcategories: sitting and reclining. To implement this distinction, APE values between 11.6° and 30° could be classified as sitting, while the values falling within the range from 30° to 73.9° could be designated as reclining. This adjustment in the classification provides a more precise representation of stationary behaviors, allowing for a more detailed examination of their potential impact on health outcomes. In interventions or individual counseling, when feasible, it may be more appropriate to use individually determined cut-points for classification of standing, sitting, reclining, and lying postures. Furthermore, in future studies, it is advisable to evaluate the effects of varying bout lengths of sitting and reclining on health outcomes using the above-mentioned cut-points.

Higher levels of moderate to vigorous PA have been observed to be associated with a lower mortality risk irrespective of the amounts of stationary time, and, in contrast, a longer stationary time is associated with a higher mortality risk in participants with low levels of moderate to vigorous PA [47]. Similarly, in the present study, a higher stationary time was associated with a lower CRF and a higher BMI, while a higher PA time in every MET bin was associated with a higher CRF and a lower BMI. Notably, in the CRF thirds and BMI categories, the difference in accumulated PA time from 7.5 METs upwards was ten-fold between the low and high thirds of CRF and categories of BMI. However, regarding the stationary behaviors, the outcomes were opposite. A longer standing time was beneficially associated with CRF and BMI, while longer lying and reclining times indicated an adverse outcome. This suggests that within stationary behaviors, the proposed specific body postures, standing, sitting, reclining, and lying, contribute differentially to health outcomes. While a longer standing time can be a consequence of a higher CRF and a lower BMI, this emphasizes the crucial role of posture measurement in device-based studies of physical behavior. Consequently, the device-based SB measurement should consider both the intensity of activity and body posture to provide a more accurate representation of their impact on health outcomes.

At present, thigh-worn devices are regarded as the gold standard for measuring SB [37,48]. Studies utilizing the thigh-worn measurements have demonstrated that less time spent in SB is strongly associated with healthier outcomes [49]. In a small sample of 13 participants, the APE method and thigh-worn device had 90% agreement between each other [20]; for example, disparities between the methods were observed when participants sat on a Swiss ball or saddle chair. Larger-scale studies are still needed to ascertain the degree of comparability between these measurement methods. 

In the present study, participants achieved an average of 258 reference vector settings per day. One hundred and twelve participants (62 female, 50 male) were excluded from the study sample as they did not meet the minimum requirement of four days with 10 daily reference vector settings. Among these excluded participants, the average number of reference vector settings was 168. These participants typically had just too many days without the required number of daily reference vector settings during the measurement week. Only five participants never achieved a single reference vector setting. The heuristic threshold of 10 settings per day may be too strict. In theory, even one reference vector setting during the whole measurement week would suffice. Further investigation is necessary to refine the criteria for reference vector settings. This includes examining the minimum daily number of settings and assessing the usability of reference vector settings from alternate days.

The APE method requires a sufficiently firm fixation of the accelerometer to the body [20]. In the present study, the accelerometer was securely positioned within a tight, elastic belt-pocket, ensuring consistent orientation relative to the body throughout the day. Further investigations are needed to develop guidelines for belt design or to establish acceptable limits for variance between reference vectors measured throughout the day. It is likely that the latter option would offer more measurable specifications for wearing the accelerometer.

Additionally, participants with poor fitness may have difficulties in reaching the MAD values of 150 mg or higher during walking, corresponding to a speed of about 3.5 km/h, which was required for setting the proper reference vector. In some target groups, e.g., in older adults, this can be problematic, and more personalized limits for reference vector settings may be required. Enhanced recognition of walking, and consequently the upright posture, could be achieved through harmonic analysis of the frequency spectra of acceleration data [50]. This approach may also mitigate the risk of falsely identifying activities other than walking as the reference posture [20].

In this study, the acceleration data were collected with a ±16 g measurement range and a 100 Hz sampling frequency. Signal saturation could adversely affect the mean values of axis-specific acceleration, and consequently the reference vector. While the peak acceleration at the hip during running may exceed 11 g [51], typically utilized measurement ranges in accelerometers, like ±8 g or ±6 g, should be sufficient for walking [52]. The sampling frequency must be sufficient to detect desired features in human activity [52]. While the APE method has been shown to operate effectively with a 52 Hz sampling frequency [39,42,43], additional research is necessary to assess its applicability with even lower sampling rates.

## 5. Conclusions

The APE method demonstrated its utility as a novel and robust framework for analyzing hip-worn data. This advancement significantly improves researchers’ capacity to thoroughly explore the epidemiology of SB and standing patterns and their causal links to various health outcomes. Ultimately, these findings are expected to play a crucial role in shaping future guidelines related to SB. Furthermore, this study highlights the need for terminology harmonization. Studies that lack a proper recognition of body posture should use the term “stationary behavior” to precisely describe the observed phenomena. The term “sedentary behavior” should be reserved for studies where the classification of SB is based on both intensity and posture. This distinction enhances clarity and accuracy of describing the physical behaviors of interest.

## Figures and Tables

**Table 1:** Number of participants in cardiorespiratory fitness (CRF) thirds and body mass index (BMI) categories broken down by age and sex.

Age Group (Years)	CRF Third	Women	Men	BMI Category	Women	Men
20–29	high	114	56	low	133	41
	mid	58	14	mid	46	32
	low	14	10	high	7	7
30–39	high	154	92	low	196	84
	mid	103	90	mid	88	83
	low	67	19	high	40	34
40–49	high	165	99	low	238	100
	mid	149	108	mid	125	134
	low	131	76	high	82	49
50–59	high	66	44	low	202	93
	mid	197	143	mid	200	185
	low	243	163	high	104	72
60–70	high	48	14	low	267	150
	mid	194	125	mid	227	243
	low	375	344	high	123	90

The first CRF tertile was 31.2 mL/kg/min for women and 34.4 mL/kg/min for men, and the second was 36.3 mL/kg/min for women and 40.4 mL/kg/min for men. BMI categories were defined as follows: low (BMI < 25 kg/m^2^), mid (25 kg/m^2^ to 30 kg/m^2^), and high (BMI > 30 kg/m^2^).

**Table 2:** The mean (SD, range) age, maximal oxygen uptake (VO_2_max), and body mass index (BMI) in cardiorespiratory fitness (CRF) thirds and BMI categories broken down by sex.

	Group		Age (Years)	VO_2_max (mL/kg/min)	BMI (kg/m^2^)
CRF thirds	women	high	36.1 (11.5, 20–69)	39.5 (2.4, 36.3–48.6)	22.0 (2.2, 16.5–32.5)
		mid	45.2 (13.7, 20–69)	33.8 (1.4, 31.2–36.3)	24.6 (2.6, 17.3–39.6)
		low	53.0 (11.9, 20–70)	26.3 (3.7, 9.9–31.2)	29.7 (4.4, 19.2–47.3)
	men	high	34.7 (10.4, 20–69)	44.0 (2.8, 40.4–53.4)	24.4 (2.8, 16.7–33.3)
		mid	45.6 (12.1, 20–69)	37.4 (1.6, 34.4–40.4)	26.4 (3.1, 14.9–35.8)
		low	54.4 (12.7, 20–70)	29.6 (4.0, 12.5–34.4)	29.0 (4.5, 19.0–48.7)
BMI categories	women	low	41.8 (14.3, 20–69)	36.8 (4.0, 19.4–48.6)	22.2 (1.8, 16.5–25.0)
		mid	47.2 (14.1, 20–70)	31.1 (3.9, 18.2–41.8)	27.1 (1.4, 25.0–30.0)
		high	50.2 (11.6, 26–70)	24.5 (4.4, 9.9–40.2)	33.7 (3.2, 30.0–47.3)
	men	low	41.7 (14.5, 20–70)	40.6 (5.4, 22.9–53.4)	23.0 (1.6, 14.9–25.0)
		mid	46.4 (13.9, 20–70)	36.3 (5.6, 16.2–51.1)	27.2 (1.4, 25.0–30.0)
		high	47.9 (13.4, 22–69)	30.9 (6.5, 12.5–46.9)	33.2 (3.4, 30.0–48.7)

The first CRF tertile was 31.2 mL/kg/min for women and 34.4 mL/kg/min for men, and the second was 36.3 mL/kg/min for women and 40.4 mL/kg/min for men. BMI categories were defined as follows: low (BMI < 25 kg/m^2^), mid (25 kg/m^2^ to 30 kg/m^2^), and high (BMI > 30 kg/m^2^).

## Data Availability

Study data are not publicly available due to identifying participant data should not be shared. Upon reasonable request, de-identified data may be available from the corresponding author.
